# Supplementary material for: A comparative analysis of microglial inducible Cre lines
Source: bioRxiv. 2023 Jan 9:2023.01.09.523268. Preprint. [Version 1] doi: 10.1101/2023.01.09.523268 (PMC9881995; doi:10.1101/2023.01.09.523268)

bioRxiv preprint doi: https://doi.org/10.1101/2023.01.09.523268; this version posted January 9, 2023. The copyright holder for this preprint (which was not certified by peer review) is the author/funder, who has granted bioRxiv a license to display the preprint in perpetuity. It is made available under aCC-BY 4.0 International license.

Table S1. DNA Primers

**Primer**	**Sequence**
C1qa^{Flox} end-point PCR, forward primer	*TGACCCTCCCAGTCTCCTGCAG*
C1qa^{Flox} end-point PCR, reverse primer	*CCCCAGGGTGCTAAAGCCCCAT*
Rosa26^{mTmG} end-point PCR, forward primer	*GCAACGTGCTGGTTATTGTG*
Rosa26^{mTmG} end-point PCR, reverse primer	*TTCTGCTGGTAGTGGTCGGCGA*
Becn1^{Flox} end-point PCR, forward primer	*GGTAGCCGCGGCCGCATTTAAA*
Becn1^{Flox} end-point PCR, reverse primer	*TGACGCCCTCTTCTGGCCTCTC*
Rosa26^{mTmG} qPCR, control, forward primer	*GCAACATCCTGGGGCACAAGCT*
Rosa26^{mTmG} qPCR, control, reverse primer	*TTCTGCTGGTAGTGGTCGGCGA*
Rosa26^{mTmG} qPCR, floxed, forward primer	*GACCGCCAAGCTGAAGGTGACC*
Rosa26^{mTmG} qPCR, floxed, reverse primer	*TGAAGCCCTCGGGGAAGGACAG*
Rosa26^{mTmG} qPCR, recombined, forward primer	*GCAACGTGCTGGTTATTGTG*
Rosa26^{mTmG} qPCR, recombined reverse primer	*GGCCATTCTCCTGTCCGTTCGC*

bioRxiv preprint doi: https://doi.org/10.1101/2023.01.09.523268; this version posted January 9, 2023. The copyright holder for this preprint (which was not certified by peer review) is the author/funder, who has granted bioRxiv a license to display the preprint in perpetuity. It is made available under aCC-BY 4.0 International license.

Supplementary Figure 1: Flow cytometry of *Rosa26^mTmG* microglia

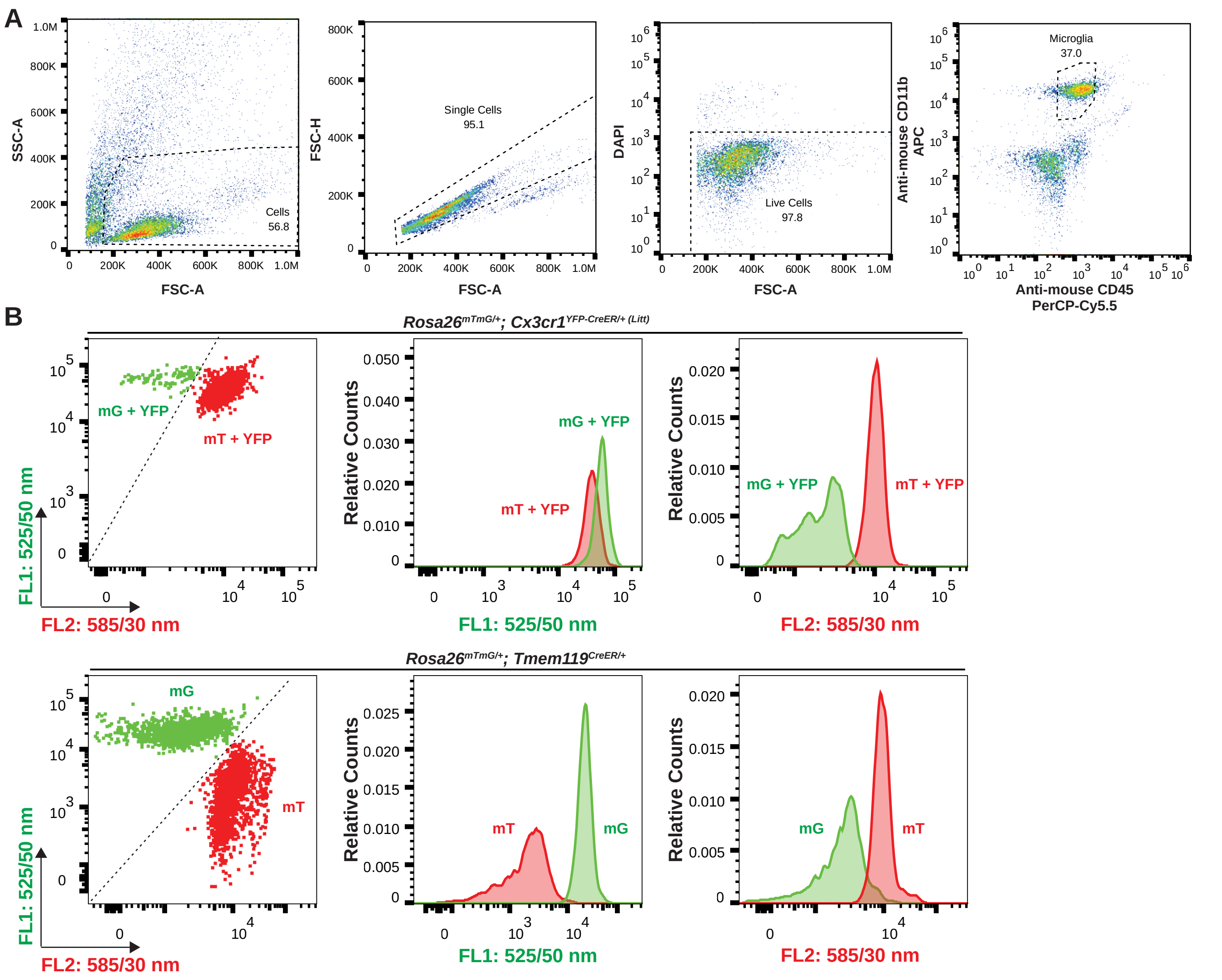

bioRxiv preprint doi: https://doi.org/10.1101/2023.01.09.523268; this version posted January 9, 2023. The copyright holder for this preprint (which was not certified by peer review) is the author/funder, who has granted bioRxiv a license to display the preprint in perpetuity. It is made available under aCC-BY 4.0 International license.

Supplementary Figure 2: Immunohistochemistry for GFP validates flow cytometry analysis of *Rosa26*^{mTmG} recombination.

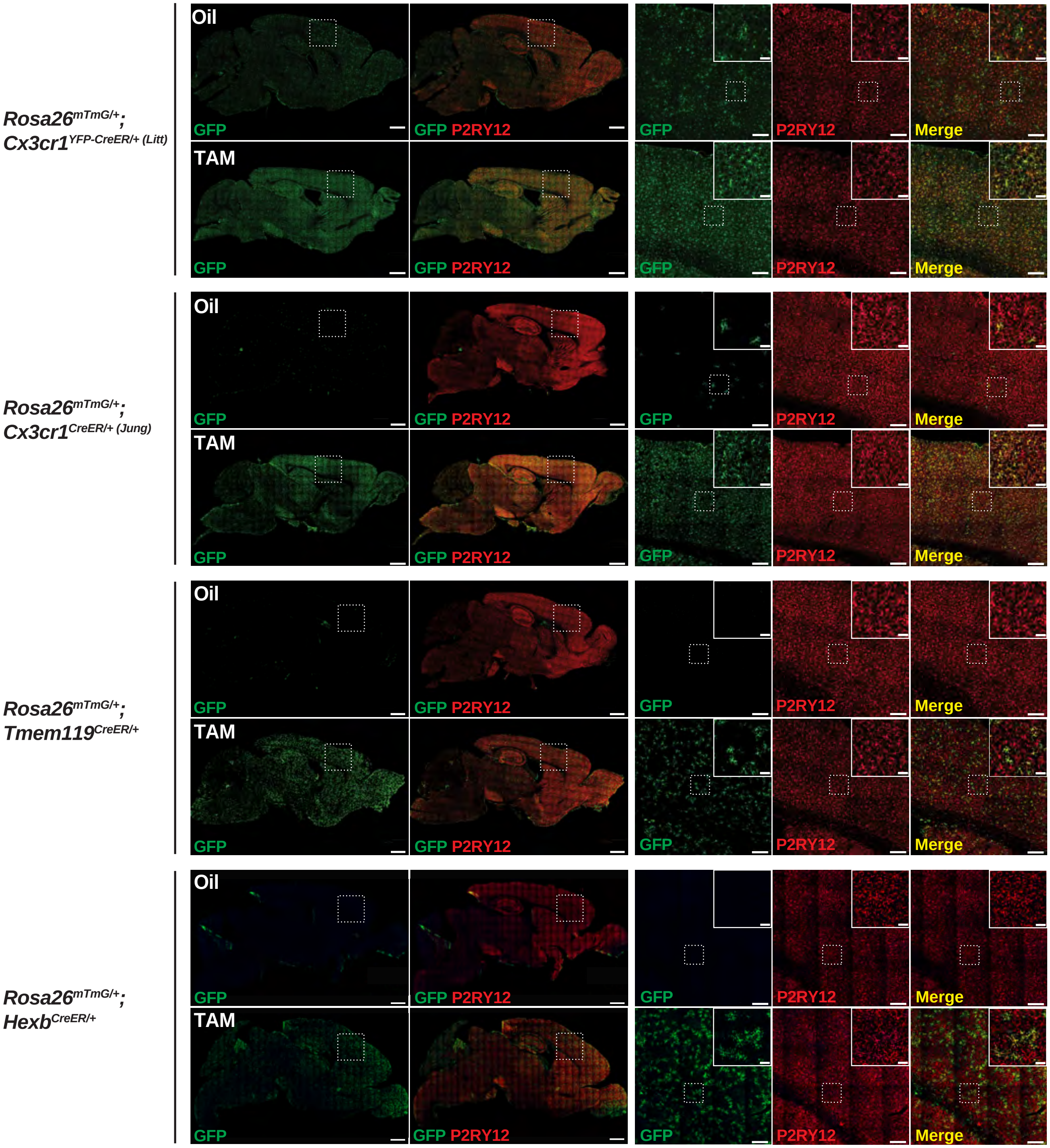

bioRxiv preprint doi: https://doi.org/10.1101/2023.01.09.523268; this version posted January 9, 2023. The copyright holder for this preprint (which was not certified by peer review) is the author/funder, who has granted bioRxiv a license to display the preprint in perpetuity. It is made available under aCC-BY 4.0 International license.

Supplementary Figure 3: Neonatal CreER activation induces loss of homeostatic microglia in *Cx3cr1*$^{YFP\text{-}CreER/+ (Litt)}$ mice

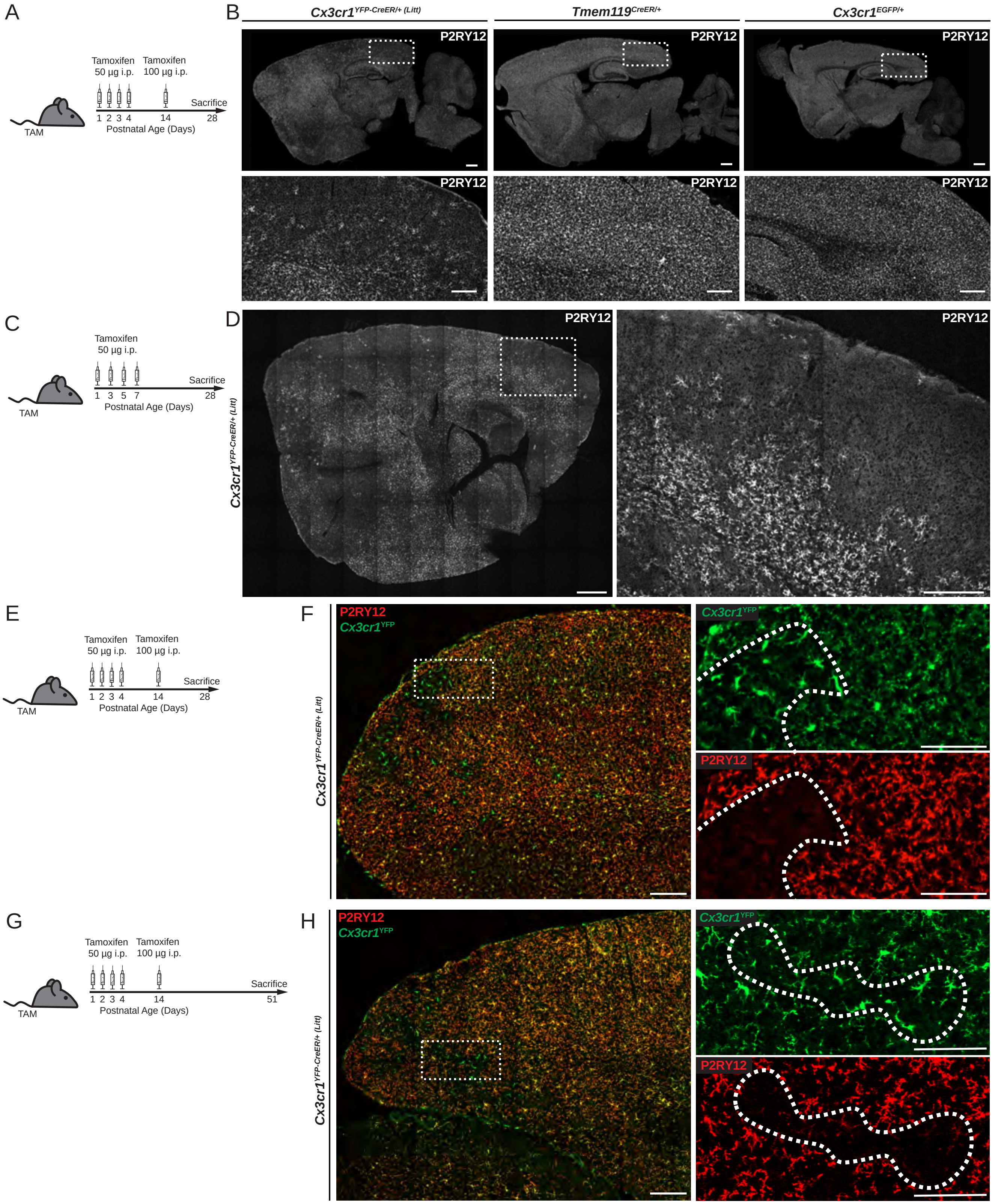

bioRxiv preprint doi: https://doi.org/10.1101/2023.01.09.523268; this version posted January 9, 2023. The copyright holder for this preprint (which was not certified by peer review) is the author/funder, who has granted bioRxiv a license to display the preprint in perpetuity. It is made available under aCC-BY 4.0 International license.

Supplementary Figure 4: *Tmem119^{CreER}* efficiently recombines short LoxP distances

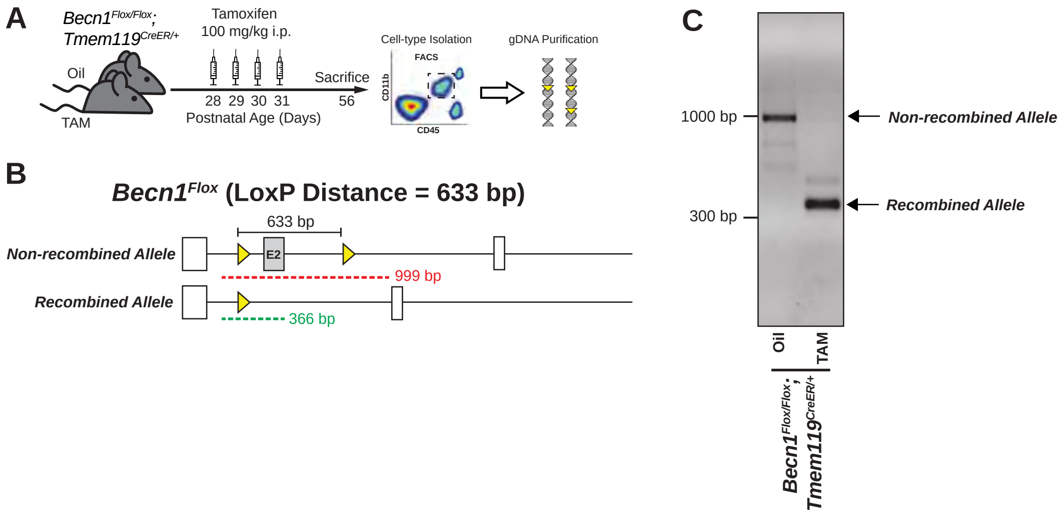

bioRxiv preprint doi: https://doi.org/10.1101/2023.01.09.523268; this version posted January 9, 2023. The copyright holder for this preprint (which was not certified by peer review) is the author/funder, who has granted bioRxiv a license to display the preprint in perpetuity. It is made available under aCC-BY 4.0 International license.

Supplementary Figure 5: Independent recombination of *Rosa26^mTmG* alleles in homozygous cells

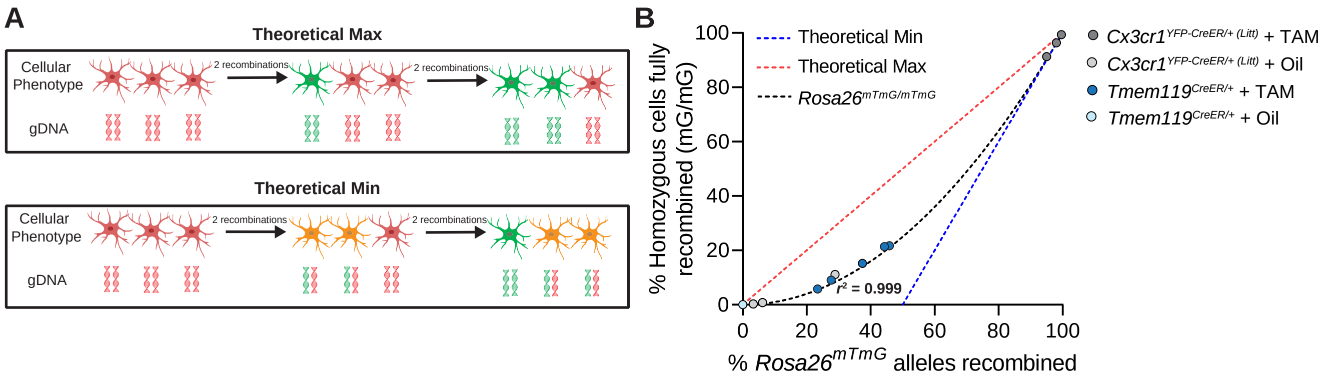

Supplement: Supplement 1 — Figure S1. Flow cytometry of Rosa26mTmG microglia. Related to Figure 1 A Plots of flow cytometry gates used for fluorescence-activated cell sorting (FACS) of microglia. B Flow cytometry analysis of recombined mGFP+ (mG) vs. non-recombined mTomato+ (mT) microglia in Rosa26mTmG/+; Cx3cr1YFP-CreER/+ (Litt) expressing YFP and Rosa26mTmG/+; Tmem119CreER/+ mice with no YFP. In both CreER lines, the recombined microglia form a distinct population identified by reduced fluorescence in the FL2 585/30 nm channel and increased fluorescence in FL1 525/50 nm channel. Figure S2. Immunohistochemistry for GFP validates flow cytometry analysis of Rosa26mTmG recombination. Related to Figure 1 Representative immunofluorescent images of brain sections from right hemisphere of oil and tamoxifen (TAM) injected mice (see also Figs. 1 and 2). Sections were immunolabeled for anti-P2RY12 (red) to identify microglia and anti-GFP (green) to identify recombined cells. The number of recombined mGFP+ microglia (white arrows) matches the results observed by flow cytometry (see Fig. 1). In the Cx3cr1YFP-CreER/+ (Litt) line, the soma of unrecombined microglia are also immunolabeled by anti-GFP due to the constitutive expression YFP. Scale bars 1 mm (full image), 200 µm (large inset), 50 µm (small inset). Figure S3. Neonatal CreER activation induces loss of homeostatic microglia in Cx3cr1YFP-CreER/+ (Litt) mice Related to Figure 3 A Timeline of neonatal tamoxifen (TAM) injection for images in (B). B Fluorescent images of brain sections from TAM injected Cx3cr1YFP-CreER/+ (Litt), Tmem119CreER/+, and Cx3cr1EGFP/+ mice, immunolabeled with anti-P2RY12. Large regions of the cortex are devoid of anti-P2RY12 immunofluorescence in Cx3cr1YFP-CreER/+ (Litt) mice, but not Tmem119CreER/+, or Cx3cr1EGFP/+ mice. Scale bars 500 µm. Insets 200 µm. C Timeline of neonatal TAM injection for images in (D). D Fluorescent images of brain sections from TAM injected Cx3cr1YFP-CreER/+ (Litt) mice, immunolabeled with anti-P2R [file NIHPP2023.01.09.523268v1-supplement-1.pdf]
